# A New Approach for Mobile Advertising Click-Through Rate Estimation Based on Deep Belief Nets

**DOI:** 10.1155/2017/7259762

**Published:** 2017-10-25

**Authors:** Jie-Hao Chen, Zi-Qian Zhao, Ji-Yun Shi, Chong Zhao

**Affiliations:** School of Software, Beijing Institute of Technology, Beijing 100081, China

## Abstract

In recent years, with the rapid development of mobile Internet and its business applications, mobile advertising Click-Through Rate (CTR) estimation has become a hot research direction in the field of computational advertising, which is used to achieve accurate advertisement delivery for the best benefits in the three-side game between media, advertisers, and audiences. Current research on the estimation of CTR mainly uses the methods and models of machine learning, such as linear model or recommendation algorithms. However, most of these methods are insufficient to extract the data features and cannot reflect the nonlinear relationship between different features. In order to solve these problems, we propose a new model based on Deep Belief Nets to predict the CTR of mobile advertising, which combines together the powerful data representation and feature extraction capability of Deep Belief Nets, with the advantage of simplicity of traditional Logistic Regression models. Based on the training dataset with the information of over 40 million mobile advertisements during a period of 10 days, our experiments show that our new model has better estimation accuracy than the classic Logistic Regression (LR) model by 5.57% and Support Vector Regression (SVR) model by 5.80%.

## 1. Introduction

With the rapid development of mobile Internet and its wide applications, mobile advertising has become one of the most successful business models in the world. To achieve the best advertisement delivery, how to best estimate the Click-Through Rate (CTR) is the key and has thus become a hot research direction in the field of computational advertising.

Generally, the display of an online advertisement can be seen as a three-side game between media, advertisers, and audiences. The advertiser purchases the chance of advertising from the media through cost-per-click (CPC), so the expected revenue of the media is decided by the Click-Through Rate (CTR) and the CPC. The formula [1] is as shown as

(1) $$\begin{array}{c} E\left(\;\text{revenue}\;\right)=\text{CTR}\times \text{CPC}. \end{array}$$

How to advertise to a specific audience group to benefit all three sides of the game process is the key problem in the field of online advertising. Inappropriate advertising will lead to the decreasing of user experience, especially in the domain of mobile advertising, with the increase of annoyance and waste of network traffic, and the advertisement will not achieve the expected effect and the media will suffer too. As a result, Click-Through Rate (CTR) estimation is the critical factor in this three-side game and has thus become a hot research direction in the field of computational advertising.

There are many models proposed by the academia and industry, which are usually based on the machine learning methods. We can divide them into three categories: linear, nonlinear, and fusion models. Chapelle [2] proposed a machine learning framework based on Logistic Regression (LR), aiming to predict the CTR for Yahoo website. Dembczyński et al. [3] adopted decision trees to discover the implicit relationships between different features and finally found out the nonlinear relationships between the predicted target and the features. He [4] developed a fusion model of LR and decision trees for the advertisement system of Facebook. The factorization based prediction method Field-Aware Factorization Machines [5] was developed by Juan et al.Tagami et al. [6] proposed a learning-to-rank approach on contextual advertising. Most of the existing prediction models have developed well and can get a satisfactory result after they are trained with massive training data whose features are chosen, built, and handled artificially, thus depending heavily on the experience and techniques of the data analysts.

Meanwhile, deep learning has become an important approach, especially after Hinton et al. [7] proposed a rapid level-by-level training process in 2006 for solving the difficulty of connecting the learning and training of the Deep Neural Network (DNN). With the help of this training process, it is now possible to train the neural network in a better way, and the neural network is widely used in multiple fields such as handwritten numeral recognition [7], 3D object recognition [8], and speech recognition [9].

The data of ads to be predicted is a kind of time-series data [10], which consists of sampled data points taken from a continuous, real-valued process over time. For the application of deep learning methods on time-series data, Chaturvedi et al. [11] presented a deep transfer learning method. The key idea is to learn high-dimensional network motifs from low-dimensional forms through a process of transfer learning. This method may greatly decrease the computational cost in the range of 25% to 600%. The approaches above provide reliable experience of applying the neural network to predict the Click-Through Rate.

Since the key features of advertisement data are not detected completely and the nonlinear relationship between different features is not fully reflected in current models, this paper proposes a mobile advertising CTR estimation fusion model based on Deep Belief Nets (DBNs). Our model combines the advantage of the powerful data representation and feature extraction capability from DBN with the advantage of simplicity of traditional Logistic Regression models. To solve the key problem of obtaining the best feature expression in machine learning, our model detects deep level features in place of simple original features and then puts it into a Logistic Regression model to predict the CTR. Numerous experimental results show that our model improves the estimation accuracy in an obvious way. It performs 5.57% and 5.80% better than the original LR model and Support Vector Regression (SVR) model in the evaluation criterion of Area under the Curves (AUC).

## 2. Deep Belief Nets

Deep Belief Nets (DBNs) are probabilistic generative models that are composed of multiple layers of stochastic, latent variables. In this part, we train our DBN by using Restricted Boltzmann Machines (RBMs) to initialize the parameters and then obtain the Backward Error Propagation (BP) Algorithm to adjust them.

### 2.1. Restricted Boltzmann Machines

**Figure 1:**
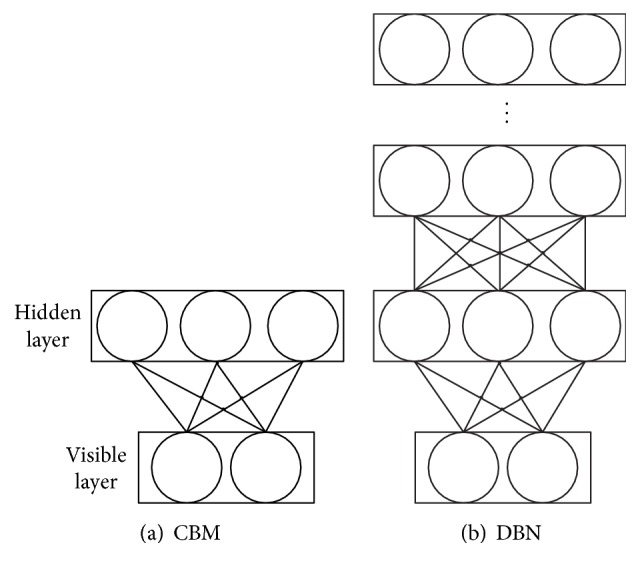
DBNs that piled up by RBMs.

Restricted Boltzmann Machine (RBM) is a two-layer undirected graph model [12], whose structure is shown in Figure 1. It consists of one visible layer and one hidden layer. There are connections between layers but no connections between units in the same layer. The units in the hidden and visible layers can be two-value units or exponential families, for example, Gaussian, Poisson, and softmax.

The tied weight and biases value define the probability distribution and energy function of the whole model, where *v* is the binary data vector of visible units and *h* is the one of hidden units, so the energy function is as follows:

(2) $$\begin{array}{c} E\left(\;v,h\;|\;\theta \;\right)=-\overset{V}{\underset{i=1}{\sum }}\;\overset{H}{\underset{j=1}{\sum }}w_{ij}v_{i}h_{j}-\overset{V}{\underset{i=1}{\sum }}b_{i}v_{i}-\overset{H}{\underset{j=1}{\sum }}a_{j}h_{j}. \end{array}$$

Here, *θ* = (*w*, *b*, *a*), *w*_*ij*_ stands for the undirected tied weight between unit *v*_*i*_ in the visible layer and unit *h*_*j*_ in the hidden layer, *b*_*i*_ and *a*_*j*_ stand for *v*_*i*_ and *h*_*j*_'s biases values, and *V* and *H* are the count of units of the visible layer and the hidden layer, respectively. When there is a specific *θ*, we can get the marginal distribution of the status vector *v* of the visible layer from the energy function:

(3) $$\begin{array}{c} p\left(\;v\;|\;\theta \;\right)=\frac{\sum_{h}e^{-E\left(v,h\right)}}{\sum_{u}\sum_{h}e^{-E\left(u,h\right)}}. \end{array}$$

The units inside one layer of RBM are not connected to each other, so when we know one specific unit in some layer, the active status of units in another layer is conditionally independent. As a result, we can calculate *p*(*v*∣*h*) and *p*(*h*∣*v*):

(4) phj=1 ∣ v,θ=σ∑i=1Vwijvi+aj,pvi=1 ∣ h,θ=σ∑j=1Hwijhj+bi,

in which *σ* = (1 + *e*^−*x*^)^−1^.

The reason why we train the RBM is to calculate the value of parameter *θ* and fit the samples. In this paper, we obtain an unsupervised training method to maximize the Log Likelihood Function *L*(*θ*) of input samples and use the stochastic gradient descent algorithm to get the partial derivative of the parameters to update the tied weight between the visible layer and the hidden layer:

(5) Lθ=ln⁡pv=ln⁡∑he−Ev,h−ln⁡∑u ∑he−Eu,h,

(6) Δwij=ε∂L∂wij=εvihjdata−vihjmodel,Δaj=ε∂Lθ∂aj=εhjdata−hjmodel.

**Figure 2:**
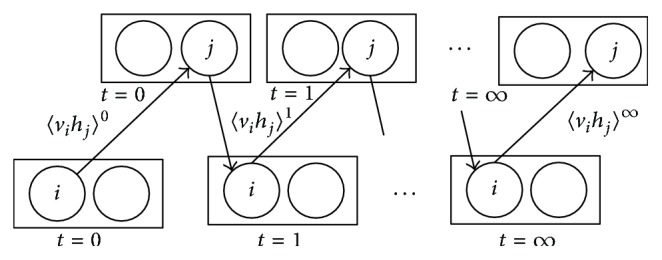
Gibbs Sampler.

In formula (6), 〈〉_data_ stands for the probability distribution of the status of the hidden layer under certain status of the visible layer, while 〈〉_model_ is the joint probability distribution of the visible layer and the hidden layer. The calculation of the joint probability distribution takes exponential time complexity, so we obtain the Contrastive Divergence Algorithm to get the approximation of the gradient by doing *t* steps of Gibbs Sampler, as shown in (7) and Figure 2:

(7) Δwij=εvihj0−vihjT,Δbi=εvi0−viT,Δaj=εhj0−hjT.

### 2.2. The Structure of Deep Belief Nets and Their Training Structure

**Figure 3:**
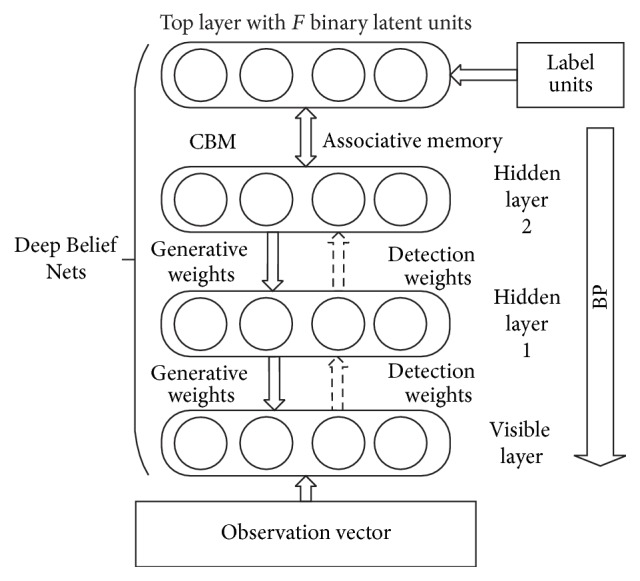
The structure of DBN.

DBN is a probabilistic generative model that contains one visible layer and multiple hidden layers, as shown in Figure 3.

The top two levels of DBN are nondirectly connected and are called the Associative Memory Layer. The rest of the layers are directly connected to one another. The downward edge is Generative Weights while the upward edge is Detection Weights. The training of DBN can be divided into two parts.

(1) To see every two layers of DBN as a single Restricted Boltzmann Machine (RBM), the hidden layer of the lower RBM is the visible layer of the upper RBM. Then, train this RBM one by one to get the initial weights of DBN.

(2) Train the DBN as a standard feedforward network and use the label data of the samples and BP [13, 14] to adjust the parameters of the model.

## 3. The Mobile Advertisement CTR Predicting Model Based on DBN

Our mobile advertisement predicting model mainly contains three steps:

(1) The pretreatment of original sample feature data.

(2) Taking the DBN to build valid features from original ones in a deep level.

(3) Taking the newly built vectors as the input of the Logistic Regression (LR) model and predicting the CTR.

**Figure 4:**
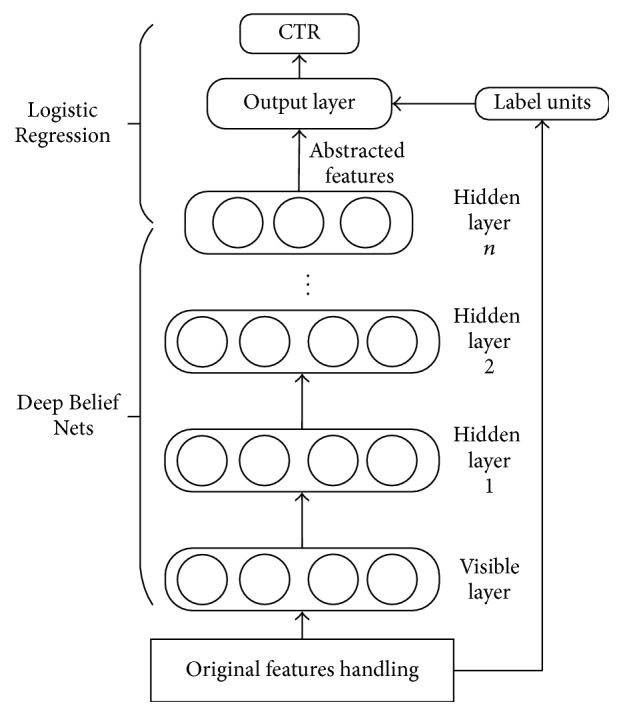
The framework of the prediction model.

The framework of the whole model is shown in Figure 4.

Compared to low level models, DBNs are known to suffer from overfitting due to the large number of parameters. And the training of these parameters needs a big amount of layer-by-layer computation, which will greatly slow down the training process when the dimension of the sample is too high. To solve this problem, we adopt the normalization method described in Section 4.3.1 on each feature in the whole dataset, which will decrease the influence of the feature values whose frequencies are extremely low.

In the structure of DBN, the activation function is a sigmoid function, which makes the best use of the nonlinear characteristics of the deeper model. Samples that are processed enter the model through the visible layer; after the detection of hidden layers, they finally become deeper detected features at the top of the model. At last, we put the deeper detected features into the LR model to get the prediction of CTR combined with label information. The output layer of the model contains one sigmoid unit, together with the units in the top hidden layer of DBN that comprises the LR model. The activation probability of the units in the output layer is as follows:

(8) $$\begin{array}{c} p\left(\;Y=1\;|\;x,\theta \;\right)=\frac{1}{1+e^{-\theta^{T}x+b}}. \end{array}$$

In formula (8), *x* is the status of units in the top layer of DBN, *θ* is the tied weight between the units in the output layer and the ones in the top layer of DBN, and *b* stands for the offset value of the output layer.

## 4. Experiments and Results

In this chapter, we design the experiments to test our model and analyze the result.

### 4.1. Experiment Environment

The hardware was an HP EliteBook 8760W WorkStation, with Intel(R) Core™ i7-2760QM 2.40 GHz CPU, 12.0 GB RAM.

The software used was Windows 10, 64 bits, MATLAB 2014Rb, DeepLearnToolbox, and JDK1.7.0.

### 4.2. Dataset

The dataset [15] we used is the Click-Through Rate Prediction Competition Dataset from Kaggle platform. The original data comes from Avazu. The training dataset contains the information of over 40 million mobile advertisements in 10 days. As a sample, we use 4,112,995 samples on the first day. We split the set into a testing dataset and a training dataset by the ratio of 9 to 1. That is to say, every single sample has 90% chance to be selected as a training set and 10% chance to be selected as a testing set. Finally, we get 412,299 samples in the test set and 3,710,696 in the training set.

**Figure 5:**
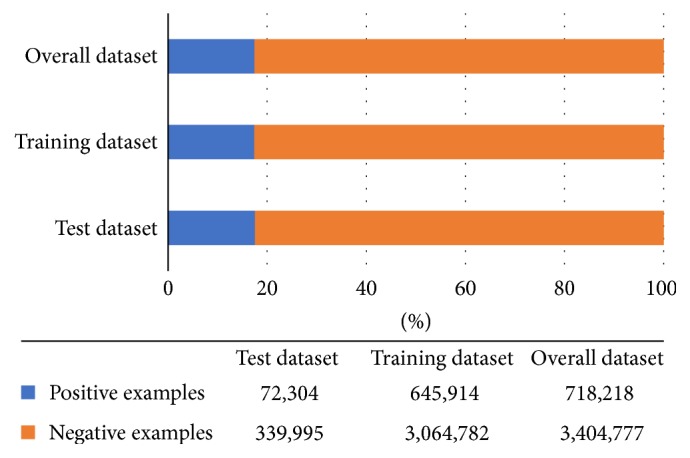
The distribution of positive and negative examples in the overall dataset, test dataset, and training dataset.

The analysis of the click result of the whole dataset is as shown in Figure 5. The positive examples are 718,218 in total while the negative examples are 3,404,777 in total, and the Click-Through Rate is around 0.174,198. Among the 412,299 test samples, the positive examples were 72,304 in total, the negative examples were 339,995, and the Click-Through Rate was about 0.175,368. In the training dataset, the positive examples are 645,914, the negative examples are 3,064,782, and the overall Click-Through Rate is around 0.174,068. The ratios of positive examples and negative examples and the Click-Through Rate are similar in the overall dataset, test dataset, and training dataset, which meet the need of our experiment.

### 4.3. The Design of the Experiment

#### 4.3.1. The Pretreatment of the Dataset

Before we begin our experiment, we first do some pretreatment on our original datasets. What is shown in Table 1 is the detailed description of the dataset; the ID is the unique identification of samples which is not going to be the input of the model; and the* click* is the label of the sample.

As for other features, first, the continuous features are divided according to the interval and then converted with digital coding to category features. Here is an example. We divide the feature* hour* into 24 different hours and the code from 0 to 23.

Second, as for the category features, we count the frequency of the different features, as shown in Table 2, which is the frequency of different values of the feature* banner_pos*.

At last, we normalize all features and use them as the input vector of DBN. There are 22 features, so the length of the input vector is 22. Take the feature banner_pos as an example; using the frequencies in Table 2, we can normalize this feature value with the following formula:

(9) $$\begin{array}{c} N_{i}=\frac{F_{i}-F_{min}}{F_{max}-F_{min}}, \end{array}$$

where *N*_*i*_ is the normalized value of the *i*th feature value *F*_*i*_ and *F*_max_ and *F*_min_ are the maximum and minimum feature values, respectively.

#### 4.3.2. Experimental Setup

To avoid overfitting, we adopt the Momentum Method and Weight-Decay Strategy to train the model. The formula which updates the tied weight in the model is as shown in

(10) Δwij=εvihjdata−vihjT−λ·wijt−1+α·Δwijt−1,

within which Δ*w*_*ij*_(*t* − 1) is the updated values of the last update. To accelerate the convergence speed of training, we adopt the minibatch method to train the model. Each batch contains 500 samples. The parameter of the training of the model is as shown in Table 3.

#### 4.3.3. Experiment

For further validation of our research result and analysis of the performance of our fusion model based on DBN, the experiment target consists of four parts.

(1) To analyze the effect of different numbers of hidden layers on the prediction of CTR.

(2) To analyze the effect of different numbers of the units in hidden layers on the prediction of CTR.

(3) To analyze the influence of different epochs on the prediction of CTR.

(4) To compare the performance of the fusion model based on DBN and other models including LR and SVR.

#### 4.3.4. Evaluating Indicator

The curve in AUC usually means the Receiver Operating Characteristic (ROC), which is usually used to evaluate the performance of a two-class classifier. While predicting a binary classification problem, it may come to four situations as shown in Table 4.

The *x*-axis of the AUC represents the false positive rate while the *y*-axis represents the true positive rate. AUC values range from 0.5 to 1, with higher value entailing better prediction performance.

### 4.4. Result Analysis

#### 4.4.1. The Influence of Different Numbers of Hidden Layers

**Figure 6:**
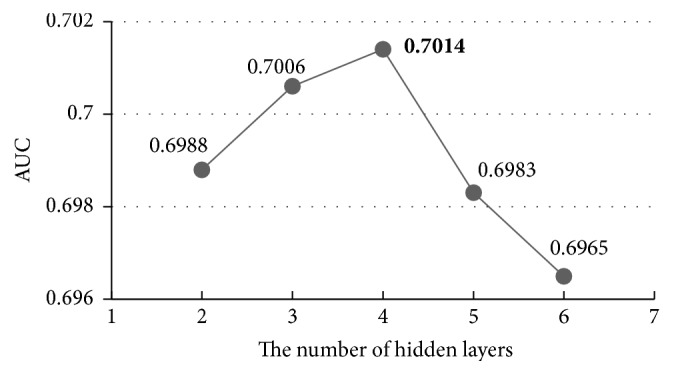
The influence of the number of hidden layers.

In this chapter, we carry out an experiment on the influence of different numbers of hidden layers in DBN. First, we set all hidden layers' numbers to 256, and the result is shown in Figure 6.

What can be seen from Figure 6 is that the AUC of the prediction result has risen apparently with the increase of the hidden layers while it reduced rapidly when the number was over 4, which is due to overfitting problems. It turns out that the more hidden layers the DBM has, the more complete the feature learning is. And for avoiding overfitting problems, we need to carry out experiments to determine the number of the hidden layers.

#### 4.4.2. The Influence of Different Numbers of Units on Hidden Layers

The number of units in hidden layers in DBN is extremely important for its great influence on the performance of the network as well as the direct reason of the overfitting problem. However, there are no universal parameter adjustment methods for this number in theory. So, in this part, we carry out experiments on the influence of this number. The set of parameters is as shown in Table 3.

**Figure 7:**
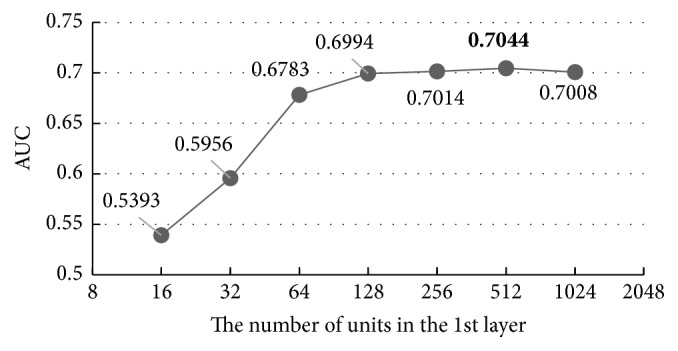
The influence of the number of the units in the first layer.

First, we investigate the influence of the units in the first layer of DBN by fixing the numbers of units in the other layers to 256 according to experiences and set the ones in the 1st as the Geometric Progression of 2^3+*i*^  (*i* ∈ *N*^+^, *i* ≤ 7). The result is shown in Figure 7.

**Figure 8:**
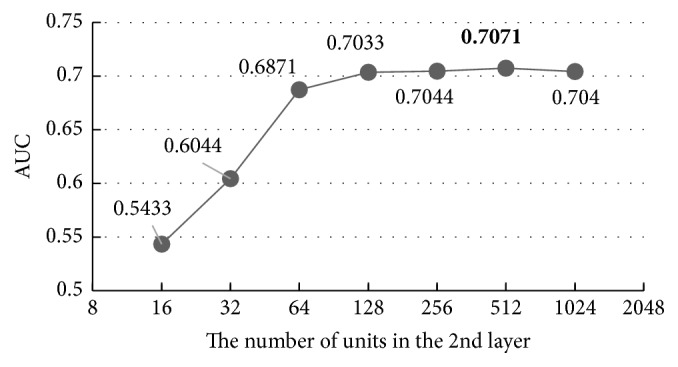
The influence of the number of the units in the second layer.

**Figure 9:**
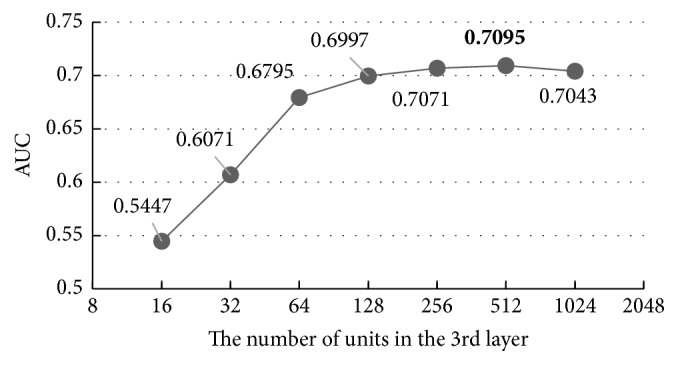
The influence of the number of the units in the third layer.

**Figure 10:**
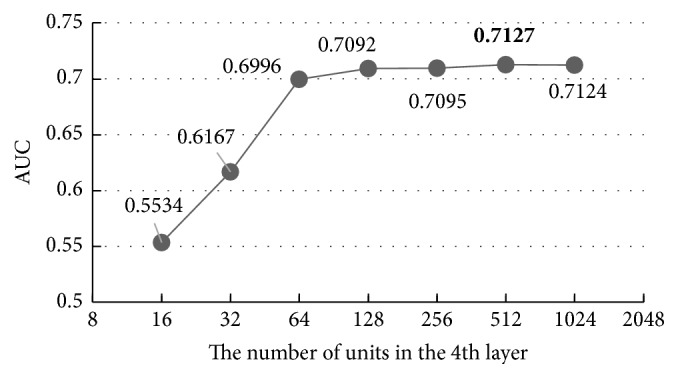
The influence of the number of the units in the fourth layer.

With the increase of units in the first hidden layer, the AUC of the results increases while the overfitting problem happens again when the unit number is more than 512, indicating that 512 is the best value, 0.43% better than the default value 256, and 30.61% better than the worst value 16. Then, we carry out the same experiment on the other hidden layers using the same parameters set in Table 3 and the results are shown in Figures 8–10.

Figures 8–10 show that all the AUC of the four layers get the highest values when the number of units is set to 512. So, we can come to a conclusion that the best values of the units in hidden layers are all 512 in our model. Too many units in the hidden layers may cause overfitting and make the model overly sensitive and unstable.

The overall accuracy of the model increased by 1.99%, from 0.6988 to 0.7127, after we adjusted the default parameters (two hidden layers with 256 units in each layer) to the best ones (four hidden layers with 512 units in each layer), which indicates the importance of parameters' adjustment clearly.

#### 4.4.3. The Influence of Different Numbers of Epochs

**Figure 11:**
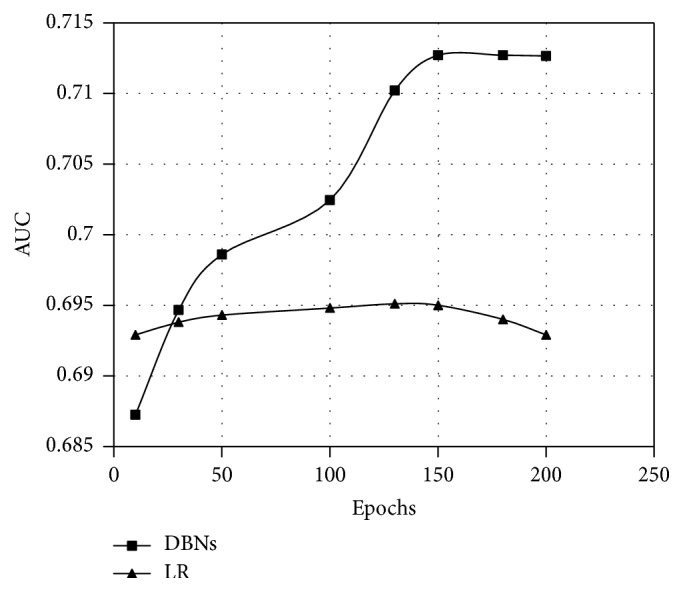
The AUC of DBNs and LR in different epochs.

Figure 11 shows the AUC of DBNs and LR in different epochs. We can see that, with the increase of epochs, DBNs get better and better performance until the epochs reach 150, and then the AUC will plateau. This reflects that the DBNs have been trained sufficiently.

We also perform an experiment on LR as contrast, the parameters of which are shown in Table 5. From Figure 11, we can see that the prediction of DBNs is better than the one of LR when the epochs are more than 30. And the performance of DBNs will not go down with the increase of epochs like LR after being trained sufficiently. This is because the LR model is a shallow model which will have an overfitting problem when the epochs become too much.

#### 4.4.4. The Comparison of DBN and Other Models

**Figure 12:**
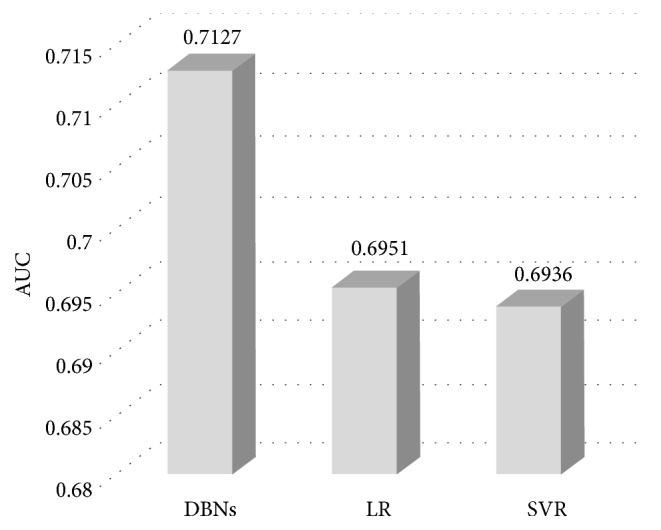
The comparison of DBN and other models.

From Figure 12, we can see that the performance of our fusion model is 5.57% better than of Logistic Regression (LR) and 5.80% better than of Support Vector Regression (SVR) in estimation accuracy.

This is because the DBN has advantages in detecting latent features which represent the essence of a sample in a better way than shallow models.

## 5. Conclusions

To solve the problem that the key features of advertisement data are not detected completely and the nonlinear relationship between different features cannot be fully reflected in traditional Click-Through Rate (CTR) estimation models, we propose a new fusion model based on Deep Belief Nets to predict the CTR of mobile advertising. Our model takes advantage of the latent feature detecting ability of the DBN and the simplicity of traditional Logistic Regression models. We also describe the derivation and training method of the model and design experiment to discuss the effect of different numbers of hidden layers as well as units in them on the prediction result. Results show that our fusion model has better performance in estimation accuracy, with a 5.57% increase compared to the classic Logistic Regression (LR) model and 5.80% increase compared to the Support Vector Regression (SVR) model.

## Figures and Tables

**Table 1:** Description of dataset.

Title	Description	Type
ID	The ID of this presentation	Category, unique
click	0: unclicked, 1: clicked	Category, 0,1
hour	Time	Continuous, 10 days, 24 hours a day
C1	Anonymous features	Continuous, 7 different values
banner_pos	The position of the ads	Category, 7 different values
site_id	The ID of the site	Category, 2,865 different values
site_domain	The domain of the site	Category, 3,394 different values
site_category	The category of the site	Category, 2 different values
app_id	The ID of the app	Category, 4,154 different values
app_domain	The domain of the app	Category, 287 different values
app_category	The category of the app	Category, 31 different values
device_id	The ID of the device	Category, 368,962 different values
device_ip	The ip of the device	Category, 1,078,153 different values
device_model	The model of the device	Category, 6,098 different values
device_type	The type of the device	Category, 4 different values
device_conn_type	The connection type of the device	Category, 4 different values
C14–C21	Anonymous features	Most categories, few continuous

**Table 2:** The frequency and normalized value of different values of the feature *banner_pos*.

Feature value	Frequency	Normalized number
0	3076333	1
1	1040891	0.338351
2	1309	0.000420
3	17	0
4	496	0.000156
5	2635	0.000851
6	1314	0.000422

**Table 3:** The set of parameters.

Parameters	Values
Units in input layer	22
Units in output layer	1
Learning rate	*ε* = 0.01
Momentum learning rate	*α* = 0.5
Weight-cost	*λ* = 0.001
Epochs	150
Unit activation function	Sigmoid
Initial weight	Gaussian distribution *N*(0,0.01^2^)
Initial biases	0
Steps in Gibbs Sampler	*T* = 1

**Table 4:** Four results of the prediction to a binary classification.

Real	Prediction	
	1	0
1	True positive, TF	False positive, FP
0	False negative, FN	True negative, TN

**Table 5:** The set of parameters.

Parameters	Values
Learning rate	*ε* = 0.01
Epochs	200
Initial weight	Gaussian distribution *N*(0,0.01^2^)
Initial biases	0

## Abbreviations

CTR: Click-Through Rate
DBN: Deep Belief Net
LR: Logistic Regression
SVR: Support Vector Regression
CPC: Cost-per-click
NN: Neural network
AUC: Area under the Curves
RBM: Restricted Boltzmann Machine
BP: Backward Error Propagation
ROC: Receiver Operating Characteristic.
